# Analysis of Amino Acid Mutations of the Foot-and-Mouth Disease Virus Serotype O Using both Heparan Sulfate and JMJD6 Receptors

**DOI:** 10.3390/v12091012

**Published:** 2020-09-10

**Authors:** Gyeongmin Lee, Ji-Hyeon Hwang, Aro Kim, Jong-Hyeon Park, Min Ja Lee, Byounghan Kim, Su-Mi Kim

**Affiliations:** Center for Foot-and-Mouth Disease Vaccine Research, Animal and Plant Quarantine Agency, 177 Hyeoksin 8-ro, Gimcheon-City, Gyeongsangbuk-do 39660, Korea; verkiss@naver.com (G.L.); jihyeonh87@korea.kr (J.-H.H.); arokim@hotmail.com (A.K.); parkjhvet@korea.kr (J.-H.P.); herb12@korea.kr (M.J.L.); kimbh61@korea.kr (B.K.)

**Keywords:** foot-and-mouth disease virus, adaptation, receptor, heparan sulfate, JMJD6, vaccine seed virus

## Abstract

Foot-and-mouth disease (FMD) is an economically devastating animal disease. Adapting the field virus to cells is critical to the vaccine production of FMD viruses (FMDV), and heparan sulfate (HS) and Jumonji C-domain-containing protein 6 (JMJD6) are alternative receptors of cell-adapted FMDV. We performed serial passages of FMDV O/SKR/Andong/2010, classified as the O/Mya-98 topotype/lineage and known as a highly virulent strain, to develop a vaccine seed virus. We traced changes in the amino acid sequences of the P1 region, plaque phenotypes, and the receptor usage of the viruses, and then structurally analyzed the mutations. VP3 H56R and D60G mutations were observed in viruses using the HS receptor and led to changes in the hydrogen bonding between VP3 56 and 60. A VP1 P208L mutation was observed in the virus using the JMJD6 receptor during cell adaptation, enabling the interaction with JMJD6 through the formation of a new hydrogen bond with JMJD6 residue 300. Furthermore, VP1 208 was near the VP1 95/96 amino acids, previously reported as critical mutations for JMJD6 receptor interactions. Thus, the mutation at VP1 208 could be critical for cell adaptation related to the JMJD6 receptor and may serve as a basis for mechanism studies on FMDV cell adaptation.

## 1. Introduction

Foot-and-mouth disease (FMD) is an acute contagious disease affecting cloven-hoofed animals, such as cows, pigs, sheep, goats, and deer. It induces fever, lameness, and vesicles on the mouth, tongue, snout, teats, and feet [1,2]. The FMD virus (FMDV) belongs to the *Aphthovirus* genus of the *Piconaviridae* family and comprises a single-stranded, plus-sense RNA genome. The virus comprises seven serotypes: A, O, C, Asia1, and southern African territories 1, 2, and 3 (SAT1, SAT2, and SAT3), of which serotype O is the most widely distributed worldwide. Adapting the field virus to cells is critical to FMDV vaccine production because the expression level of integrin receptors, known as the wildtype virus receptor, is low in suspension baby hamster kidney (BHK-21) cells commonly used for FMD inactivated vaccine production [3]. Therefore, several mutations related to the change in receptor usage were reported in the vaccine seed viruses [4,5,6,7]. Among other mutations, the H56R mutation in VP3 is well known to induce an ionic interaction between Arg and the sulfate group of heparan sulfate (HS) as the secondary receptor, and it is the most critical amino acid residue for BHK-21 suspension cell cultures [8]. In addition, 55–60 amino acids of VP3, 133–138 amino acids of VP2, and 195–197 amino acids of VP1 have been reported to form the canonical HS-binding site [7,9] and several mutations were suggested to be related to structural interactions with HS in previous studies [10]. However, all the mutations detected in vaccine seed viruses may not be related to interactions with the HS receptor because the CPE was observed in both Chinese hamster ovary-K1 (CHO-K1) cells containing HS and Chinese hamster ovary-677 (CHO-677) cells not containing HS infected with some cell-adapted viruses.

Several reports have demonstrated that the Jumonji C-domain-containing protein 6 (JMJD6), expressed in CHO-677 cells, plays a role in broadening cell tropism as the third receptor of FMDV [11,12,13,14,15]. JMJD6 is also expressed in FMDV-permissive cells like BHK-21 and porcine kidney (LFBK) cells [16]. JMJD6 was identified as a cell membrane phosphatidylserine receptor for the recognition and clearance of the apoptotic cells [17]. It has been also implicated that the phosphatidylserine receptor could have the role in the infection of enveloped viruses [18,19]. E95K and S96L VP1 mutations in FMDV serotype A were found in soluble integrin αvβ6 resistant and CHO-677 cell permissive virus (A-SIR #42) and reported as the critical amino acid residues to interact with JMJD6, considered as the third receptor in a previous study [13,15]. However, few mutation analysis studies of cell-adapted FMDV related to the JMJD6 receptor have been reported, although more data on amino acid mutations predicted to interact with the JMJD6 receptor in various FMDV serotypes are needed to reveal the interaction mechanism between the FMDV capsid and the JMJD6 receptor.

In this study, we performed serial passages of FMDV O/SKR/Andong/2010, classified as O/Mya-98 topotype/lineage and highly virulent, to make a vaccine strain. [20]. Further, we traced the change in amino acid sequences of the P1 region, plaque phenotypes, and viral receptor usage during the serial passages. We observed that specific amino acids were changed during the change of receptor usage of the viruses. In addition, we analyzed the relative locations of the amino acid mutations, such as VP3 56, VP3 60, and VP1 208 and the change in hydrogen bonds and interactions with JMJD6 in the FMDV structure.

## 2. Materials and Methods

### 2.1. Cells and Viruses

Baby hamster kidney (BHK)-21 adherent cells, porcine kidney (LFBK) cells, black goat kidney (BGK) cells, and BGK-integrin-β6 cells were cultured in Dulbecco’s modified Eagle’s medium (Thermo Fisher Scientific, Waltham, MA, USA). Chinese hamster ovary-K1 (CHO-K1) cells (ATCC CCL-61, Manassas, VA, USA) and Chinese hamster ovary-677 (CHO-677, pgsD-677) cells (ATCC CRL-2244, Manassas, VA, USA) were maintained in D-MEM/F12 (Thermo Fisher Scientific, Waltham, MA, USA) culture medium. The media were supplemented with 10% fetal bovine serum (pH 7.4), and the cells were grown at 37 °C in a 5% CO_2_ incubator. The LFBK cells were supplied by Plum Island Animal Disease Center (Orient, NY, USA) [21]. BGK and BGK-integrin-β6 cells stably expressing bovine integrin-β6 were established in the Animal and Plant Quarantine Agency (APQA) [22]. BHK-21 suspension cells were cultured in ProVERO-1 serum-free medium (Lonza, Basel, Switzerland) with dextran sulfate (Sigma-Aldrich, MO, USA), L-glutamine (Thermo Fisher Scientific, Waltham, MA, USA), pluronic F-68 non-ionic surfactant (Thermo Fisher Scientific, Waltham, MA, USA), and HyClone cell boost five supplement (Fisher Scientific, Hampton, NH, USA) at 37 °C and 110 rpm in a 5% CO_2_ shaking incubator. The BHK-21 suspension cells were established by APQA and the Korea Research Institute of Bioscience and Biotechnology, Republic of Korea. FMDV O/SKR/Andong/2010 (Genbank accession No. KC503937) was isolated in 2010 by APQA in Andong city, province of Gyeonsangbuk-do, Republic of Korea. The viral titers were measured and calculated using the Reed and Muench method at a 50% tissue culture infective dose (TCID_50_) [23].

### 2.2. Serial Passaging of FMDV

The viruses serially passaged for cell adaptation of the field virus were prepared: (1) five times in LFBK cells; (2) five times in LFBK cells and six times in adherent BHK-21 cells; (3) five times in LFBK cells, six times in adherent BHK-21 cells, and once in suspension BHK-21 cells; and (4) five times in LFBK cells, six times in adherent BHK-21 cells, and twice in suspension BHK-21 cells. The virus was infected at an MOI (multiplicity of infection) of 0.001, and the virus cultured media were harvested at 16 h post-infection in serial passages. FMDV infection and culturing were performed in a Bio-Safety Level 3 facility in APQA.

### 2.3. Plaque-Forming Assay

Plaque-forming assay of serially passaged viruses was performed to observe plaque phenotypes [24]. BHK-21 adherent cells in 6-well plates were infected with the O/SKR/Andong/2010 viruses adsorbed for 1 h. After which, the melted SeaPlaque agarose (Lonza, Basel, Switzerland) was overlaid and incubated at 37 °C for 72 h. The cells were fixed with 4% formalin and stained with 0.2% crystal violet, and the plaque phenotype was observed.

### 2.4. Genome Amplification, Nucleotide Sequencing, and Amino Acid Sequence Alignment

Viral RNA was extracted from 100 μL of the supernatant of serially passaged O/SKR/Andong/2010 viruses using MagNA Pure 96 system (Roche, Basel, Switzerland). The RNA was treated using a one-step PCR inhibitor removal kit (ZYMO Research, CA, USA), and single-stranded cDNA was prepared by reverse transcription using an oligo-(dT)_18_ primer and SuperScript II reverse transcriptase (Thermo Fisher Scientific, Waltham, MA, USA). The P1 (VP4, VP2, VP3, and VP1) region was PCR amplified using Phusion High-Fidelity DNA polymerase (Thermo Fisher Scientific, Waltham, MA, USA), forward primer (5′-GGAGCCGGGCAATCCAGT-3′), and reverse primer (5′-CTGCTTTACAGGTGCCACT-3′), and purified using ExoSAP–IT Express PCR Cleanup Reagent (Thermo Fisher Scientific, Waltham, MA, USA). Nucleotide sequencing was performed by Macrogen Inc. (Seoul, Korea) using an ABI 3730xl DNA analyzer (Applied Biosystems, Foster city, CA, USA). The amino acid sequences of the P1 region were edited using the CLC Main Workbench (version 6.9.1, Qiagen Bioinformatics, Redwood city, CA, USA) and aligned using CLUSTAL W software (Version 1.8) [25].

### 2.5. Structural Analysis and the Molecular Docking Model

The locations of mutated amino acid residues were marked in the pentameric structure using the Mol star (Mol*) three-dimensional (3D) viewer [26]. The crystal structure of O PanAsia (PDB accession no. 5NE4) was a template for modeling the capsid protein (protomeric subunit) of FMDV O/SKR/Andong/2010 using SWISS-MODEL [27]. Indication of mutated amino acid residue locations in protomeric subunits and hydrogen bond analysis were performed using the Pymol molecular graphics system (Version 2.3.4, Schrodinger LLC, New York, NY, USA). Molecular docking of human JMJD6 (PDB accession no. 3LD8, Chain A) and VP1 of L5B5S2 or the field O/SKR/Andong/2010 virus was performed on the ClusPro protein–protein docking web server [15,28].

## 3. Results

### 3.1. Change in the Plaque Phenotypes and the FMDV O/SKR/Andong/2010 Receptor by Serial Passaging

**Figure 1:**
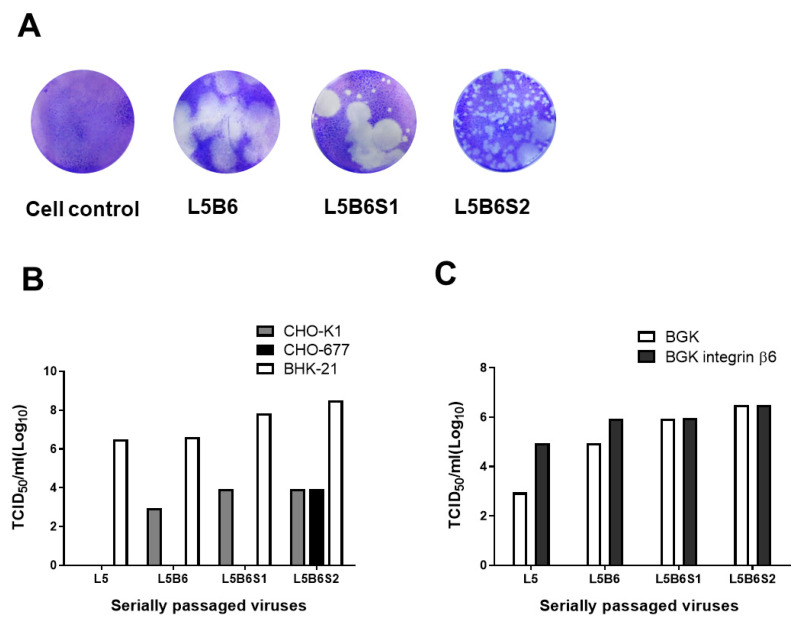
The change in receptor usage of the O/SKR/Andong/2010 virus following serial passaging. The O/SKR/Andong/2010 virus used in this study was serially passaged five times in porcine kidney (LFBK) cells (L5); five times in LFBK cells and six times in adherent baby hamster kidney (BHK)-21 cells (L5B6); five times in LFBK cells, six times in adherent BHK-21 cells and once in suspension BHK-21 cells (L5B6S1); or five times in LFBK cells, six times in adherent BHK-21 cells and two times in suspension BHK-21 cells (L5B6S2). The plaque phenotypes were compared using plaque forming assay (**A**). Virus titrations were performed in Chinese hamster ovary (CHO)-K1, CHO-677 and adherent BHK-21 cells to test the viral usage of the heparan sulfate (HS) receptor (**B**) and were performed in black goat kidney (BGK) and BGK-integrin-β6 cells to test the dependency of the integrin receptor (**C**).

To detect the change in plaque phenotypes and receptor usage of the virus by serial passaging, plaque assays with BHK-21 adherent cells and virus titration with various cells were performed (Figure 1). CHO-K1 cells were used to test the HS dependence of FMDV, and CHO-677 cells not expressing HS and integrins were used to test the JMJD6 dependence of FMDV as an alternative receptor [13,29]. FMDV permissive cells, BGK, and BGK-integrin-β6 were used to observe the change in dependency on the integrin αvβ6 receptor in serially passaged O/SKR/Andong/2010 viruses [22]. The trend of the plaque phenotypes changed from large to small during the process of adaptation to BHK-21 cells (Figure 1A). The L5B6 virus displayed large plaques, and most L5B6S1 virus plaques were large. However, only a few L5B6S2 plaques were large. The virus titer increased gradually in BHK-21 cells with serial passaging (Figure 1B). The L5 virus had no viral titer in CHO-K1 and CHO-677 cells, hence, the second HS and third JMJD6 receptors were not used. However, the L5B6S1 virus could use HS as a receptor, and the L6B6S2 virus could use both HS and JMJD6 as receptors. We observed that the enhancement in viral titer by the integrin αvβ6 receptor, which is the major receptor of the field virus, decreased gradually and disappeared in L5B6S1 and L5B6S2 (Figure 1C).

### 3.2. Amino Acid Mutation Analysis of P1 Regions of O/SKR/Andong/2010 by Serial Passaging

Amino acid mutations were not observed in the P1 region of the L5 virus compared to those of the field virus (Table 1). His-to-Arg substitution at amino acid position 56 (H56R) of VP3 and Asp-to-Gly at position 60 (D60G) were commonly observed in L5B6, L5B6S1, and L5B6S2 which used the HS receptor. Furthermore, His-to-Tyr substitution at amino acid 34 position (H34Y) of VP2 was observed in L5B6S1 and L5B6S2. Pro-to-Leu substitution at amino acid position 208 (-4 position of the C terminus, P208L) of VP1 was observed in L5B6S2 which used the JMJD6 and HS receptors. Ala-to-Gly substitution at amino acid position 205 of VP2 was observed in only L5B6 and disappeared.

### 3.3. Three-Dimensional (3D) Structural Analysis of Mutated Amino Acid Residues by Serial Passaging

**Figure 2:**
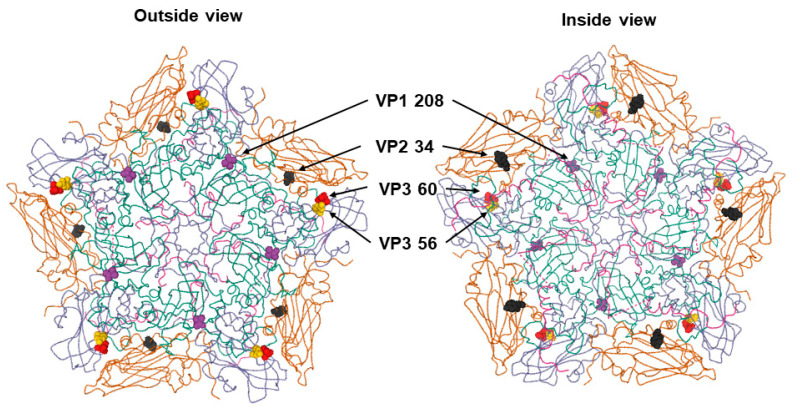
Relative locations of the mutated amino acid residues found in the cell-adapted O/SKR/Andong/2010 virus on the three-dimensional structural model of the foot-and-mouth disease virus (FMDV) serotype O pentamer. The positions of mutated amino acid residues of the cell-adapted virus (L5B6S2), including VP1 208 (purple), VP2 34 (black), VP3 56 (yellow), and VP3 60 (red) are indicated in the outer and inner schematic views of a pentameric subunit (O PanAsia, PDB accession no. 5NE4). The protein subunits of VP1, VP2, VP3, and VP4 are colored cyan, yellow, violet, and pink, respectively.

The locations of mutated amino acid residues were mapped in the 3D structure of the FMDV serotype O pentamer model (Figure 2). Amino acid position 208 of VP1 (VP1 208), and positions 56 and 60 of VP3 (VP3 56 and VP3 60) were located outside the capsid, while position 34 of VP2 (VP2 34) was located inside the capsid. VP3 60 was near VP3 56, a critical residue for binding with the HS receptor. VP1 208 was distinct from VP3 56 and 60 and closer to the five-fold axis in the capsid than VP3 56 or VP3 60.

### 3.4. Hydrogen Bond Analysis of L5B6S2 by Amino Acid Mutations

**Figure 3:**
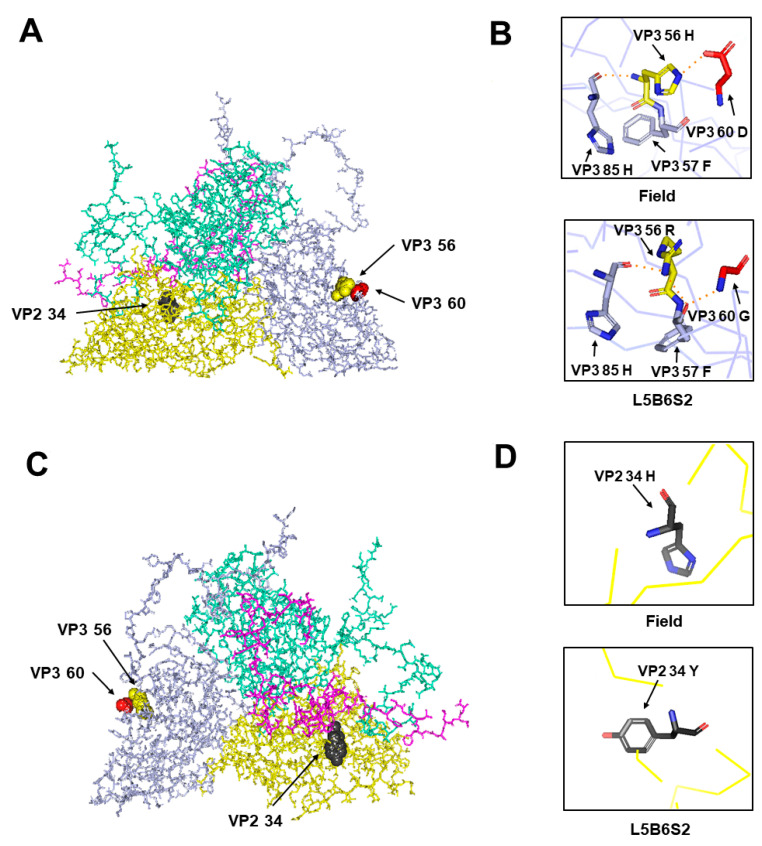
Hydrogen bond analysis of mutated amino acid residues. The locations of mutated amino acid residues, such as VP3 56, VP3 60 (**A**) (outer view), and VP2 34 (**C**) (inner view) are designated in the 3D structure of the protomeric subunit of the cell-adapted (L5B6S2) virus. The protein subunits of VP1, VP2, VP3, and VP4 are colored cyan, yellow, violet, and pink, respectively. Hydrogen bonds between the mutated residues, including VP3 56, VP3 60 (**B**), and VP2 34 (**D**) and other residues in the protomeric subunit of the field or cell-adapted (L5B6S2) virus were analyzed. The orange dotted lines represent the hydrogen bonds.

The H56R and D60G of VP3 led to a change in hydrogen bonds (Figure 3). VP3 60 was near VP3 56 (Figure 3A) in the protomer model of the O/SKR/Andong/2010 virus. The hydrogen bond between VP3 56 H and VP3 60 D was disrupted, and new hydrogen bonds between VP3 57 F and VP3 60 G or VP3 56 R were created (Figure 3B). The hydrogen bond between VP3 56 H and VP3 85H remained unchanged. The amino acid at VP2 34 was near the VP4 protein inside the protomer (Figure 3C). No hydrogen bond associated with VP2 34 was observed in the protomer subunit, and only the positively charged amino acid, His (H), was substituted with a neutral-charged amino acid, Tyr (Y) (Figure 3D).

### 3.5. Predicted Interaction between the L5B6S2 Virus and the JMJD6 Protein by the VP1 208 Amino Acid Mutation

**Figure 4:**
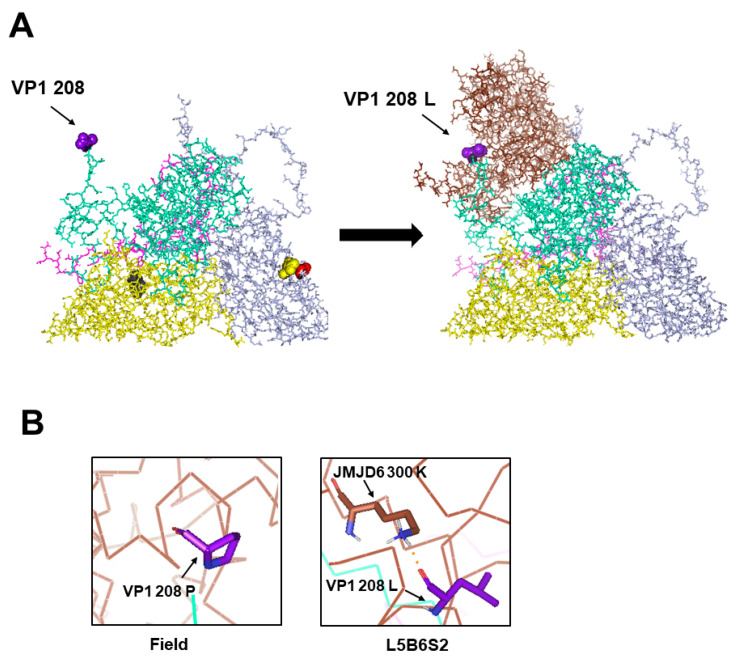
Predicted interaction between the protomer subunit of the O/SKR/Andong/2010 virus and the JMJD6 protein with the VP1 208 amino acid mutation. (**A**) The VP1 208 amino acid residue is in the outer schematic view of the protomeric subunit of the cell-adapted (L5B6S2) virus on the left. The molecular docking model shows the protomeric subunit of the cell-adapted (L5B6S2) virus and the JMJD6 protein mediated by the VP1 208 L residue on the right. VP1, VP2, VP3, and VP4 of FMDV, and JMJD6 are colored cyan, yellow, violet, pink, and brown, respectively, in the outer schematic view. (**B**) The hydrogen bond analysis was represented between the 208 amino acid of VP1 and the amino acid of JMJD6 in the field and cell-adapted virus (L5B6S2). The orange dotted lines represent the hydrogen bonds.

**Figure 5:**
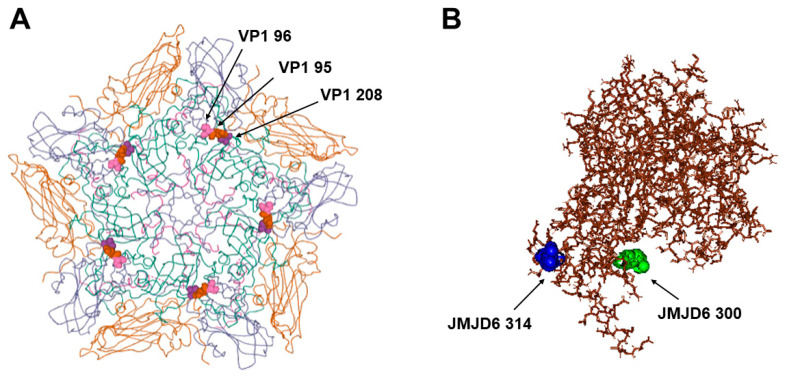
Relative locations of amino acid residues reported in a previous study and this study on the three-dimensional structural model of the FMDV pentamer and the JMJD6 protein. (**A**) The amino acid residues at positions VP1 95 and 96 [15], and VP1 208 (this study) are indicated in the outer schematic view of a pentameric subunit (O PanAsia, PDB accession no. 5NE4). The protein subunits of VP1, VP2, VP3, and VP4 are colored cyan, yellow, violet, and pink, respectively. (**B**) Amino acid residues 314 [15] and 300 (this study) are indicated in the JMJD6 protein.

We predicted the interaction between VP1 208 and the JMJD6 protein using the docking model (Figure 4). The substitution of VP1 208 Pro (P) with Leu (L) enabled the attachment of L5B6S2 FMDV with the JMJD6 protein (Figure 4A). The new hydrogen bond between VP1 208 L of FMDV and 300 K of JMJD6 was created by the VP1 208 P to L mutation (Figure 4B). In addition, we checked the relative location of VP1 208 and the mutation, VP1 95/VP1 96, reported as a critical amino acid for interaction with the JMJD6 receptor [15]. We observed that the VP1 208 position was close to the VP1 95 or the VP1 96 position, which was reported to be the critical amino acid in interaction with the JMJD6 receptor (Figure 5A). Furthermore, JMJD6 300 residue predicted as interacting with VP1 208 L was also near JMJD6 314 residue, and was observed to form a hydrogen bond with VP1 95/VP1 96 of FMDV in a previous study (Figure 5B) [15].

## 4. Discussion

Cellular adaptation of the FMDV field isolate is essential for its culture in baby hamster kidney (BHK)-21 suspension cells, which are eventually used to produce FMD vaccine on a large scale; furthermore, the change in receptor usage can affect several characteristics of FMDV. It was reported that cell-adapted FMDV using the HS receptor has lower pathogenesis, and the virus using the JMJD6 receptor was not virulent in aerosol-infected cattle [30,31]. It was also reported that thermostability and the entry pathway of FMDV could be affected by the change in receptor usage [30,32]. In this study, we analyzed receptor usage, plaque phenotypes, and amino acid mutations during the developing process of FMDV O/Mya-98 topotype/lineage vaccine seed virus (Figure 1 and Table 1). We observed that the viral titer was increased during cell adaptation of the related HS receptor, and it increased more after the usage of the JMJD6 receptor (L5B6S2) in BHK-21 cells. The phenomenon was also observed in other vaccine seed viruses in our laboratory (data not shown). Depending on integrin αvβ6, the first receptor decreased in the L5B6 virus and disappeared in L5B6S1 and L5B6S2 viruses (Figure 1). Therefore, we concluded that L5B6 selected HS or integrin, L5B6S1 selected HS and not integrin, and L5B6S2 selected HS or JMJD6 and not integrin. Therefore, we predicted that both VP3 mutations, H56R and D60G, would interact with the HS receptor and the VP1 P208L mutation would interact with the JMJD6 receptor (Table 1). In this study, we observed large plaques in the O/SKR/Andong/2010 viruses using the HS receptor. However, several small plaques and a few large plaques were observed in O/SKR/Andong/2010 viruses using JMJD6 (L5B6S2) (Figure 1). This suggested that O/SKR/Andong/2010 viruses using the HS receptor had a large plaque phenotype, whereas viruses using the JMJD6 receptor had a small plaque phenotype. In a previous report, small plaques were observed in the cell-adapted O1 Campos with VP3 56R in BHK-21 cells [5]. However, another recent study reported that the O1 Campos with VP3 56R had a similar plaque size as O1 Campos with VP3 56H and not small plaques in BHK-21 cells [30]. Virus populations might be mixed following serial passages, and cell tropism becomes diversified. Therefore, the data on nucleotide sequencing and plaque phenotype in this study could provide representative results of viral populations.

We conclude that the amino acid mutation of the VP3 60 position also plays a critical role in the interaction with the HS receptor by changing the hydrogen bond related to the amino acid at the VP3 56 position. The substitution of the positively charged amino acid (VP3 H56R) is well known as the most important residue for interaction with the HS receptor [5,33,34]. It is in the βB “knob” of VP3, which forms one wall of the HS-binding depression, and the positively charged VP3 56R could ionically interact with the negatively charged sulfate groups [6,7]. The combination mutation of VP3 H56R and VP3 D60G has been reported in several cell-adapted FMDV serotype O strains, such as O1 Campos, O1 Lausanne, and O1 BFS 1860 [5,35,36,37]. Simultaneous amino acid mutations at the VP3 56 and 60 positions were commonly observed in all the L5B6, L5B6S1, and L5B6S2 viruses using the HS receptor in this study (Figure 1 and Figure 2). Furthermore, the hydrogen bond mediated VP3 57 amino acid followed the mutations of the VP3 56 and 60 amino acids, and the negatively charged Asp changed to non-polar Gly in the VP3 60 position (Figure 3). The changes could promote the interaction between the viral capsid and the HS receptor. It has been observed that hydrogen bonding is important to the interaction receptors and ligands, and the change in hydrogen bond is related to viral adaptation [38,39,40]. Furthermore, VP3 60 was near the VP3 56 position (Figure 2). Neighboring residues, such as VP2 134 and VP3 56, have been reported as critical for the interaction with HS; it is known that VP3 55–60, VP2 133–138, and VP1 195–197 amino acid residues form the HS-binding pocket [5,7].

In this study, we observed the VP1 P208L mutation in the O/SKR/Andong/2010 virus, using the JMJD6 receptor, which suggested that the VP1 208 mutation was related with the usage of the JMJD6 receptor. This is supported by several data. VP1 208 is very close to the C-terminus of the VP1 region (C-terminus of VP1 −4 amino acid position) and a surface-exposed amino acid residue, not overlapping with the HS-binding pocket (Figure 2). Therefore, we analyzed the VP1 208 mutation in relation to interaction with the JMJD6 receptor and observed that the binding and hydrogen bonding with the JMJD6 receptor appeared to be established by the VP1 208 Pro-to-Leu mutation (Figure 4). A previous study reported proposed JMJD6 as an alternative receptor, and the VP1 95K/96L mutation enabled hydrogen bond formation with the JMJD6 receptor [15,41]. The VP1 208 amino acid was near the VP1 95 and 96 amino acid in the pentameric structure of FMDV serotype O and both JMJD6 314 (interacting with VP1 95K) and JMJD6 300. The amino acids to form hydrogen bonds with FMDV residues were in the C-terminal of JMJD6 (Figure 5) [17]. JMJD6 is a membrane-bound intracellular nuclear protein that recognizes and clears apoptotic cells [17,42,43]. Therefore, the C-terminal region containing residues 300 and 314 of the JMJD6 gene is predicted as an extracellular region downstream of the transmembrane domain. Furthermore, the VP1 208 residue mutation may be related to the findings of a previous report in which the C-terminal region of VP1 (positions 201 to 211 in serotype O) was involved in virus attachment to cells [44]. Mutations in the C-terminal region of VP1 (positions 201 to 211) have been reported in cell-adapted FMDV in recent studies [41,45]. The C-terminal region of VP1 (positions 201 to 211) had no interaction with HS, and it remains unclear if the region mediated binding with the receptors [6]. Therefore, we suggest that the interaction between the C-terminal region of VP1 (position 201 to 211) and the JMJD6 receptor needs to be tested. Considering that the VP1 208 residue is critical in the FMDV antigenic site one and is near the five-fold axis of the virion, the accessibility of the position to receptors might be high [7,46,47]. Therefore, the possibility that the mutation decreases the level of neutralizing antibody titers cannot be overruled. In this study, we observed the mutation in the cell adaptation of field virus. However, to show the role of a single mutation of VP1 208 on the interaction with the JMJD6 receptor, reverse genetics (infectious cDNA clone with a point mutation of VP1 208 amino acid) should be used. The usage of the JMJD6 receptor of FMDV was always observed after the usage of the HS receptor during the cell adaptation process of FMDV. Therefore, the amino acid substitution for the usage of JMJD6 might not be independent on the amino acid substitutions related to HS.

In conclusion, we analyzed critical amino acid mutations for the O/Mya-98 FMDV vaccine seed virus and the P208L of VP1, which is thought to be critical for interaction with the JMJD6 receptor. Several reports exist on cell adaptation in relation to the HS receptor; however, only a few studies report the relationship between the amino acid mutations and the JMJD6 receptor. Therefore, we comprehensively analyzed the vaccine seed virus to provide detailed information about the link between FMDV and the cellular receptor. In this study, we provided data on the relationship between new amino acid mutations and the JMJD6 receptor. The results obtained may be useful for future studies of the mechanism of the interaction between JMJD6 and the FMDV capsid, and provide important information for the design of recombinant FMDV vaccines. In further studies, confirmation assays using the FMDV infectious clone with site-directed mutations and detailed characterization of the thermostability, acid stability and pathogenesis will be conducted.

## Figures and Tables

**Table 1:** Amino acid mutations of the P1 region of the O/SKR/Andong/2010 virus following serial passaging.

Serially Passaged Viruses	VP2		VP3		VP1
	Amino Acid Positions ^e^				
	34	205	56	60	208
Wildtype	H	A	H	D	P
L5 ^a^	**·**	**·**	**·**	**·**	**·**
L5B6 ^b^	**·**	G	R	G	**·**
L5B6S1 ^c^	Y	**·**	R	G	**·**
L5B6S2 ^d^	Y	**·**	R	G	L

^a^ O/SKR/Andong/2010 field virus was serially passaged five times in LFBK cells (L5); ^b^ O/SKR/Andong/2010 field virus was serially passaged five times in LFBK cells and six times in adherent BHK-21 cells (L5B6).; ^c^ O/SKR/Andong/2010 field virus was serially passaged five times in LFBK cells, six times in adherent BHK-21 cells, and once in BHK-21 cell (L5B6S1) suspension. ^d^ O/SKR/Andong/2010 field virus was serially passaged five times in LFBK cells, six times in adherent BHK-21 cells, and two times in BHK-21 cell (L5B6S2) suspension. ^e^ Identical amino acid sequences to those of the O/SKR/Andong/2010 field virus are represented by dots.

## References

[1] Alexandersen S., Zhang Z., Donaldson A.I., Garland A.J. (2003). The pathogenesis and diagnosis of foot-and-mouth disease. J. Comp. Pathol..

[2] Moraes M.P., de Los Santos T., Koster M., Turecek T., Wang H., Andreyev V.G., Grubman M.J. (2007). Enhanced antiviral activity against foot-and-mouth disease virus by a combination of type I and II porcine interferons. J. Virol..

[3] Amadori M., Volpe G., Defilippi P., Berneri C. (1997). Phenotypic features of BHK-21 cells used for production of foot-and-mouth disease vaccine. Biologicals.

[4] Neff S., Sá-Carvalho D., Rieder E., Mason P.W., Blystone S.D., Brown E.J., Baxt B. (1998). Foot-and-mouth disease virus virulent for cattle utilizes the integrin alpha(v)beta3 as its receptor. J. Virol..

[5] Sa-Carvalho D., Rieder E., Baxt B., Rodarte R., Tanuri A., Mason P.W. (1997). Tissue culture adaptation of foot-and-mouth disease virus selects viruses that bind to heparin and are attenuated in cattle. J. Virol..

[6] Fry E.E., Lea S.M., Jackson T., Newman J.W., Ellard F.M., Blakemore W.E., Abu-Ghazaleh R., Samuel A., King A.M., Stuart D.I. (1999). The structure and function of a foot-and-mouth disease virus-oligosaccharide receptor complex. EMBO J..

[7] Fry E.E., Newman J.W.I., Curry S., Najjam S., Jackson T., Blakemore W., Lea S.M., Miller L., Burman A., King A.M.Q. (2005). Structure of Foot-and-mouth disease virus serotype A10 61 alone and complexed with oligosaccharide receptor: Receptor conservation in the face of antigenic variation. J. Gen. Virol..

[8] Han S.C., Guo H.C., Sun S.Q. (2015). Three-dimensional structure of foot-and-mouth disease virus and its biological functions. Arch. Virol..

[9] Chamberlain K., Fowler V.L., Barnett P.V., Gold S., Wadsworth J., Knowles N.J., Jackson T. (2015). Identification of a novel cell culture adaptation site on the capsid of foot-and-mouth disease virus. J. Gen. Virol..

[10] Dill V., Eschbaumer M. (2020). Cell culture propagation of foot-and-mouth disease virus: Adaptive amino acid substitutions in structural proteins and their functional implications. Virus Genes.

[11] Xu X., Nagarajan H., Lewis N.E., Pan S., Cai Z., Liu X., Chen W., Xie M., Wang W., Hammond S. (2011). The genomic sequence of the Chinese hamster ovary (CHO)-K1 cell line. Nat. Biotechnol..

[12] Lidholt K., Weinke J.L., Kiser C.S., Lugemwa F.N., Bame K.J., Cheifetz S., Massagué J., Lindahl U., Esko J.D. (1992). A single mutation affects both N-acetylglucosaminyltransferase and glucuronosyltransferase activities in a Chinese hamster ovary cell mutant defective in heparan sulfate biosynthesis. Proc. Natl. Acad. Sci. USA.

[13] Lawrence P., LaRocco M., Baxt B., Rieder E. (2013). Examination of soluble integrin resistant mutants of foot-and-mouth disease virus. Virol. J..

[14] Zhao Q., Pacheco J.M., Mason P.W. (2003). Evaluation of genetically engineered derivatives of a Chinese strain of foot-and-mouth disease virus reveals a novel cell-binding site which functions in cell culture and in animals. J. Virol..

[15] Lawrence P., Rai D., Conderino J.S., Uddowla S., Rieder E. (2016). Role of Jumonji C-domain containing protein 6 (JMJD6) in infectivity of foot-and-mouth disease virus. Virology.

[16] Lawrence P., Conderino J.S., Rieder E. (2014). Redistribution of demethylated RNA helicase A during foot-and-mouth disease virus infection: Role of Jumonji C-domain containing protein 6 in RHA demethylation. Virology.

[17] Lawrence P., Rieder E. (2017). Insights into Jumonji C-domain containing protein 6 (JMJD6): A multifactorial role in foot-and-mouth disease virus replication in cells. Virus Genes.

[18] Moller-Tank S., Maury W. (2014). Phosphatidylserine receptors: Enhancers of enveloped virus entry and infection. Virology.

[19] Morizono K., Chen I.S. (2014). Role of phosphatidylserine receptors in enveloped virus infection. J. Virol..

[20] Pacheco J.M., Lee K.N., Eschbaumer M., Bishop E.A., Hartwig E.J., Pauszek S.J., Smoliga G.R., Kim S.M., Park J.H., Ko Y.J. (2016). Evaluation of Infectivity, Virulence and Transmission of FDMV Field Strains of Serotypes O and a Isolated in 2010 from Outbreaks in the Republic of Korea. PLoS ONE.

[21] LaRocco M., Krug P.W., Kramer E., Ahmed Z., Pacheco J.M., Duque H., Baxt B., Rodriguez L.L. (2015). Correction for LaRocco et al., A Continuous Bovine Kidney Cell Line Constitutively Expressing Bovine αVβ6 Integrin Has Increased Susceptibility to Foot-and-Mouth Disease Virus. J. Clin. Microbiol..

[22] Kim S.M., Kim S.K., Lee K.N., Park J.H., Kim B. (2020). Stable expression of bovine integrin beta 6 increases susceptibility of goat kidney cell line to foot-and-mouth disease virus. J. Bacteriol. Virol..

[23] Reed L.J., Muench H. (1938). A simple method of estimating fifty percent end points. Am. J. Hyg..

[24] Lewis A.M., Rowe W.P. (1970). Isolation of two plaque variants from the adenovirus type 2-simian virus 40 hybrid population which differ in their efficiency in yielding simian virus 40. J. Virol..

[25] Thompson J.D., Higgins D.G., Gibson T.J. (1994). CLUSTAL W: Improving the sensitivity of progressive multiple sequence alignment through sequence weighting, position-specific gap penalties and weight matrix choice. Nucleic Acids Res..

[26] Sehnal D., Rose A.S., Koča J., Burley S.K., Velankar S. (2018). Mol*: Towards a common library and tools for web molecular graphics. Workshop on Molecular Graphics and Visual Analysis of Molecular Data.

[27] Keedy D.A., Georgiev I., Triplett E.B., Donald B.R., Richardson D.C., Richardson J.S. (2012). The role of local backrub motions in evolved and designed mutations. PLoS Comput. Biol..

[28] Comeau S.R., Kozakov D., Brenke R., Shen Y., Beglov D., Vajda S. (2007). ClusPro: Performance in CAPRI rounds 6-11 and the new server. Proteins.

[29] Baranowski E., Ruiz-Jarabo C.M., Sevilla N., Andreu D., Beck E., Domingo E. (2000). Cell recognition by foot-and-mouth disease virus that lacks the RGD integrin-binding motif: Flexibility in aphthovirus receptor usage. J. Virol..

[30] Borca M.V., Pacheco J.M., Holinka L.G., Carrillo C., Hartwig E., Garriga D., Kramer E., Rodriguez L., Piccone M.E. (2012). Role of arginine-56 within the structural protein VP3 of foot-and-mouth disease virus (FMDV) O1 Campos in virus virulence. Virology.

[31] Lawrence P., Pacheco J., Stenfeldt C., Arzt J., Rai D.K., Rieder E. (2016). Pathogenesis and micro-anatomic characterization of a cell-adapted mutant foot-and-mouth disease virus in cattle: Impact of the Jumonji C-domain containing protein 6 (JMJD6) and route of inoculation. Virology.

[32] Martín-Acebes M.A., González-Magaldi M., Sandvig K., Sobrino F., Armas-Portela R. (2007). Productive entry of type C foot-and-mouth disease virus into susceptible cultured cells requires clathrin and is dependent on the presence of plasma membrane cholesterol. Virology.

[33] Mohapatra J.K., Pandey L.K., Rai D.K., Das B., Rodriguez L.L., Rout M., Subramaniam S., Sanyal A., Rieder E., Pattnaik B. (2015). Cell culture adaptation mutations in foot-and-mouth disease virus serotype A capsid proteins: Implications for receptor interactions. J. Gen. Virol..

[34] Morioka K., Fukai K., Ohashi S., Sakamoto K., Tsuda T., Yoshida K. (2008). Comparison of the characters of the plaque-purified viruses from foot-and-mouth disease virus O/JPN/2000. J. Vet. Med. Sci..

[35] Forss S., Strebel K., Beck E., Schaller H. (1984). Nucleotide sequence and genome organization of foot-and-mouth disease virus. Nucleic Acids Res..

[36] Kitson J.D., McCahon D., Belsham G.J. (1990). Sequence analysis of monoclonal antibody resistant mutants of type O foot and mouth disease virus: Evidence for the involvement of the three surface exposed capsid proteins in four antigenic sites. Virology.

[37] Pfaff E., Thiel H.J., Beck E., Strohmaier K., Schaller H. (1988). Analysis of neutralizing epitopes on foot-and-mouth disease virus. J. Virol..

[38] Hadži D., Kidrič J., Koller J., Mavri J. (1990). The role of hydrogen bonding in drug-receptor interactions. J. Mol. Struct..

[39] Luo J., Deng L., Ding X., Quan L., Wu A., Jiang T. (2017). Hydrogen Bond Variations of Influenza A Viruses During Adaptation in Human. Sci. Rep..

[40] Wang Y., Liu M., Gao J. (2020). Enhanced receptor binding of SARS-CoV-2 through networks of hydrogen-bonding and hydrophobic interactions. Proc. Natl. Acad. Sci. USA.

[41] Dill V., Hoffmann B., Zimmer A., Beer M., Eschbaumer M. (2018). Influence of cell type and cell culture media on the propagation of foot-and-mouth disease virus with regard to vaccine quality. Virol. J..

[42] Fadok V.A., Bratton D.L., Rose D.M., Pearson A., Ezekewitz R.A., Henson P.M. (2000). A receptor for phosphatidylserine-specific clearance of apoptotic cells. Nature.

[43] Fadok V.A., de Cathelineau A., Daleke D.L., Henson P.M., Bratton D.L. (2001). Loss of phospholipid asymmetry and surface exposure of phosphatidylserine is required for phagocytosis of apoptotic cells by macrophages and fibroblasts. J. Biol. Chem..

[44] Fox G., Parry N.R., Barnett P.V., McGinn B., Rowlands D.J., Brown F. (1989). The cell attachment site on foot-and-mouth disease virus includes the amino acid sequence RGD (arginine-glycine-aspartic acid). J. Gen. Virol..

[45] Dill V., Hoffmann B., Zimmer A., Beer M., Eschbaumer M. (2017). Adaption of FMDV Asia-1 to Suspension Culture: Cell Resistance Is Overcome by Virus Capsid Alterations. Viruses.

[46] Xie Q.C., McCahon D., Crowther J.R., Belsham G.J., McCullough K.C. (1987). Neutralization of foot-and-mouth disease virus can be mediated through any of at least three separate antigenic sites. J. Gen. Virol..

[47] Berryman S., Clark S., Kakker N.K., Silk R., Seago J., Wadsworth J., Chamberlain K., Knowles N.J., Jackson T. (2013). Positively charged residues at the five-fold symmetry axis of cell culture-adapted foot-and-mouth disease virus permit novel receptor interactions. J. Virol..

